# Skills for communicating severe acute respiratory syndrome-coronavirus-2 result to patients and/or relatives

**DOI:** 10.4102/safp.v63i1.5221

**Published:** 2021-05-13

**Authors:** Oladele V. Adeniyi, Dominique K. Kayembe

**Affiliations:** 1 Department of Family Medicine, Faculty of Health Sciences, Walter Sisulu University, Mthatha, South Africa; 2 Department of Family Medicine, Faculty of Health Sciences, Cecilia Makiwane Hospital, East London, South Africa

**Keywords:** bad news, COVID-19, notification, structured model, telephonic consultation

## Abstract

Clinicians notify positive results of the severe acute respiratory syndrome coronavirus-2 polymerase chain reaction to patients and/or relatives, whilst short message service (SMS) has been adopted as a means of disseminating negative results. Therefore, clinicians should be adequately equipped to provide telephonic consultation whilst delivering a positive test result to patients. The news of the coronavirus disease 2019 (COVID-19) test result often invokes fear of impending death in patients, especially the elderlies and those with comorbidities. In addition, several survivors have reported persistent symptoms and COVID-19-related stigma, which precludes them from immediate re-integration into their workplaces. Consequently, COVID-19 results are perceived as bad news by the members of the public. This article justifies why COVID-19 test results are bad news and also discusses the notification steps to follow when delivering COVID-19 results, whilst also addressing patients’ immediate concerns. The article concludes by highlighting an important safety net for COVID-19 patients and the attending clinician.

## Introduction

With the increasing incidence of caronavirus disease 2019 (COVID-19) cases at the population level,^1^ many health facilities can no longer admit all patients under investigations (PUIs), especially those with mild disease. In addition, there is significant pressure on bed spaces for admission of PUIs. The implication thereof is that patients with mild disease would be managed as outpatients and being in self-quarantine, whilst waiting for the results of the severe acute respiratory syndrome-coronavirus-2 (SARS-CoV-2). Clinicians are expected to share the results with the patients, which is often performed telephonically or face to face. The process of communicating a COVID-19 result may put undue pressure on the clinician who may not be experienced in breaking unpleasant (bad) news.

Bad news is often considered in the context of events that drastically and negatively change a person’s views of his or her future.^2^ Common examples of bad news include sudden death of a family member, diagnosis of incurable diseases (human immunodeficiency virus [HIV], cancers), highly infectious diseases such as Ebola virus, Yellow fever, viral haemorrhagic fever and many others. This article focuses on two important questions, namely why the diagnosis of COVID-19 disease should be considered in a similar context as the conditions mentioned here, and what are the steps to follow in communicating the results of COVID-19 to patients.

Members of the general public perceive themselves as vulnerable to the virus with the possibility of fatal consequences, if infected. People believe that the virus is highly contagious and is associated with significant morbidity and mortality.^3,4,5,6,7^ This perception is supported by evidence on the transmission dynamics, epidemiological data and lack of effective vaccines and antiviral therapies. The person-to-person transmission of the SARS-CoV-2 via contact, droplets and being airborne through aerosol generating procedures is a serious public health concern.^8^ The high reproductive number (*R*_0_ = 2.2–2.5) coupled with ‘super spreaders’ create a pandemic, which has spread over 200 countries worldwide.^1^ This is compounded by the fact that asymptomatic individuals, as well as patients in the pre-symptomatic phase, often spread the virus.^9,10,11^ This is associated with high risk of transmission of COVID-19 within clusters amongst household members.^12^ Patients often have concerns about the risk of infecting their family members at the time of accessing an oropharyngeal swab test. Thus, the concerns about their health and that of their loved ones increase patients’ anxiety upon receiving the diagnosis of COVID-19 disease. This often invokes feelings of guilt, blame and anger. In addition, survivors of COVID-19 disease share their experiences of sleep difficulties, paranoia of acquiring the virus from someone else, fear, social stigma and discrimination and the overall negative impact on their daily activities.^13^ This further heightens anxiety.

Moreover, the increasing trend of mortality caused by COVID-19 at the community level, nationally and globally has become a cause for panic for patients. The COVID-2019 related mortalities as on 30 August 2020 was 847 087 (out of 25 189 744 cases) globally and 13 981 (out of 622 551 cases) in South Africa.^1^ Clustering of deaths within families has also been reported in the literature.^3,12^ Although, the fatality rate for COVID-19 cases ranges from 0.5% to 4%, it has killed more people than the combined deaths attributed to the 2003 severe acute respiratory syndrome and the Middle-East respiratory syndrome, which killed 774 people (10% case-fatality rate) and 858 people (34% case-fatality rate), respectively.^14^ The COVID-19 mortality demonstrates dose-response effect with ageing: 0.2% in young adults, 3.6% (60–69 years), 8% (70–79 years) and 15% (80 years and above).^15,16^ In addition, there are significant associations with other comorbidities, such as controlled diabetes mellitus (hazard ratio [HR] = 4.65, 95% confidence interval [CI]: 3.19–6.79); poorly controlled diabetes (9%) (HR = 13.02, 95% CI: 10.06–16.87); current tuberculosis (HR = 2.58, 95% CI: 1.53–4.37) and HIV (HR = 2.75, 95% CI: 2.09–3.61).^16^

In addition to the fear of death, survivors of COVID-19 disease have reported prolonged symptoms (71.4%) after recovery, beyond 60 days post-isolation. Common symptoms that persist include, but are not limited to, fatigue, dry cough, loss of taste and smell, headache, chest pain and difficulty in breathing.^17^ More so, there is an increase in reports of COVID-19-related stigma amongst the survivors.^13,18^ Anecdotally, many patients have been discriminated against within the work place, after diagnosis of COVID-19. Overall, the experiences of the patients with COVID-19 have been considered unpleasant and thus, have now become bad news to newly diagnosed patients. As such, there is serious panic and anxiety associated with the diagnosis of COVID-19 in individuals infected with the virus.

In order for clinicians to be able to communicate this unpleasant news (COVID-19 result) to patients, a specific set of skills is required. Many doctors lack the skills to manage the immediate concerns, fears, misperceptions, misinformation and any other queries that may be raised by the patients upon delivery of the bad news.^19^ In addition, some doctors are limited by their experiences and knowledge of the disease or fear of death. It is, therefore, important that doctors must follow a structured approach in implementing this arduous task. The approach to breaking bad news has evolved and several models of communicating bad news have been reported in the literature. The SPIKES^20^ (Setting; Perception; Invitation or Information; Knowledge; Empathy; Summarise or Strategise) and ABCDE^21^ models are very popular models, which can be adapted to fit virtually all sets of conditions. The steps described in this article incorporated the natural history of COVID-19,^12^ South African guideline for the clinical management of suspected or confirmed cases, Version 5 (24 August 2020),^22^ steps for breaking bad news by Bob Mash and Blitz^23^ and SPIKES and ABCDE models.^20,21,23^

## Steps for delivering the COVID-19 results to patients and/or relatives

### Step 1: Preparation for notification

#### What?

It is very important to know and have all the relevant information to share with the patient in addition to the positive result. It is expected that you would have gone through the history of the current illness; onset of the first symptom(s), any comorbidities of the patient that may increase his or her risk for severe COVID-19 disease, conditions for continued self-isolation at home and others. Also, you should have estimated how many days from the onset of first symptom to the day you are sharing the result (symptomatic cases) or from the day the swab was taken to the day of sharing the result in cases of high risk asymptomatic contacts.^22^

Based on the turn-around time of the result, which is variable (ranges from 1 day to 10 days or longer, depending on the level of facility or geographic distance to the main laboratory), some patients may have qualified for de-isolation.

- Mild cases: De-isolate after 10 days (earlier guideline recommended 14 days) from the onset of the first symptom.
- Asymptomatic cases: De-isolate after 10 days (previously 14 days) from the day the swab was taken.
- Severe cases: De-isolate after 10 days (previously 14 days) from the time when the patient became stable (taken off oxygen).^22^

#### Where?

Evidence suggests that 81% of symptomatic individuals will have mild disease.^8^ As such, they would be managed as outpatients, either in self-isolation in their homes or in designated isolation centres. As such, it is important to know where the patient is located beforehand in order to arrange for privacy.

#### Who?

Given that patients often have questions, concerns and fears, which must be addressed upon delivering of the results,^3,4,5^ the golden rule is only doctors and nurses who are knowledgeable about COVID-19 should deliver the results.

### Step 2: Setting up the scene

It is very important to create an appropriate context (time and physical space to ensure privacy and confidentiality) for the notification of the result. You want to minimise interruptions during the consultation (either telephonically or face to face). You must devote time to the process to ensure that you can listen and understand the wishes, reactions and concerns of the patients, which are often diverse.^23^ It is also important to establish that the patient has some minutes to spare, and he or she is in a relatively comfortable place and condition before disclosing the COVID-19 result. Firstly, you need to assess the current condition of the patient and enquire about what has happened since the swab for COVID-19 was taken. Any form of deterioration in the clinical condition of the patient will require formal hospital consultation.

Next, assess the patient’s perception of COVID-19. What does the patient know about COVID-19; how serious it can be, any risk of sudden change in his or her condition and what must be done if this happens? It is important to link the patient’s understanding of COVID-19 with the realities of the existing knowledge in the literature. Crucially, you must be able to assess the emotional level (tone of voice, terms used or avoided) of the patient from his or her responses. You must also assess the verbal (words) and non-verbal (hand movements, body posture, facial expressions) cues. In telephonic consultation, non-verbal cues may be difficult to glean, but certain expressions can communicate the emotions of the patients such as breathing depth, breaks in-between speech and variations in tone of voice can be picked up. The goal of this observation is to assess the level of anxiety of the patient. However, clinicians should not judge any of the responses of the patient, but rather add to the important information being provided for the further management of the patient.

Lastly, find out how much the patient wants to know (this applies to patients with severe to critical disease). It should be noticed that the most critical question in delivering bad news is the extent of information the patient wants to know. Some patients, at the hospital admission stage, want to know if they will die from COVID-19! The prerogative for you as clinician, therefore, is not only to provide enough information to assist the patient but also to watch closely and stop when the patient signals that he has heard enough. Often, it is difficult to know when a patient has had enough information about a condition. Hence, it is advisable to ask the patient directly whether he or she wants to be given the prognosis of the condition.

### Step 3: Sharing the COVID-19 result

Firstly, you must anticipate varied responses and reactions upon delivery of the result; you must be ready for this. Give such patients an opportunity to express their feelings to the result of COVID-19 in any way they choose.

It is important to reinforce the correct understanding of COVID-19, which will set the tone for educating patients further. Lastly, attempt to change patients’ understanding in small steps whilst observing their responses.

Give a warning shot about the unpleasant news about to be delivered. For example:

- ‘I’m afraid that this result is not what we expected’…

Give information in bite size chunks in a simple, honest and comprehensible manner, preferably in the patient’s home language:

- ‘Your COVID-19 result is positive’ …

Check reception of the result and clarify understanding of the information:

- ‘Which means that all the symptoms you are experiencing were caused by this virus …’

### Step 4: Addressing the patient’s emotional responses

It is important for clinicians to know that a patient’s emotional responses may range from silence (shut-down) to distress. Observe and listen to the patient’s verbalised emotions. Give time for him or her to express his or her emotions. Encourage and acknowledge a patient’s emotions, concerns and fears about the disease:

‘What are you feeling or thinking right now?’

Demonstrate empathy and acceptance. Specifically elicit questions and concerns if none are forthcoming.

Check for his or her understanding of risk of spread of the infection to other contacts (besides those recorded at the time of the test within the household, co-workers or casual contacts). There may be need to prepare an additional list of contacts he or she may have been in contact with during isolation. Reinforce his or her understanding of the vital importance of infection control and prevention measures whilst in isolation and the importance of compliance with these measures.

‘Hand washing, wearing of a mask and social distancing from everyone at home’ …

### Step 5: Sharing further management plans

#### Clinical condition

Given that a large proportion of patients receiving swab tests for COVID-19 are managed on an outpatient basis and some may be experiencing clinical deterioration at the time of sharing of the result, it is important to assess the severity of the patient and the need for continued outpatient care. The key determinant in this regard is the level of breathing difficulty and whether the patient can continue self-isolation at home or in a designated facility. Often, patients are provided with an information leaflet detailing what they need to do if their conditions change whilst at home. Some patients in designated facilities might have access to oxygen saturation monitoring, testing of blood sugar levels and overall evaluation of their general condition. Any sudden deterioration in the condition of the patient will warrant emergency care. Whilst consulting patients with the result, this is an important opportunity to assess the clinical condition of the patient; presenting symptoms may be improving, deteriorating or persisting and new symptoms may have emerged. Depending on the findings, such a patient may need to continue with self-isolation (if improving in condition), call emergency medical services for Emergency Department (ED) (if experiencing a sudden deterioration) or book a clinical appointment (if experiencing persistent symptoms).

Make sure that you assist the patient as much as possible. Share the plans of assistance for all the immediate contacts of the patient. All contacts should be placed on self-quarantine. These high-risk contacts should be offered a naso-oropharyngeal swab test in accordance with the guideline.^22^

### Step 6: Closing

In closing, it is important to reinforce the probable risk of deterioration in the condition if the patient is still within 10 days of the onset of the symptoms. Clinicians must avoid giving false hope about the prognosis of the patients, especially when dealing with COVID-19 in elderly patients and those with comorbidities. Sudden deterioration and death have been reported in patients with COVID-19.^16^ Clinicians must provide a safety net for themselves and their patients. This can be achieved by issuing patient information sheet. The COVID-19 information sheet comprises the natural history, risk factors for and symptoms of deterioration in the condition of patients, and what to do and where to seek help if patient observes clinical deterioration. Also, it is important to identify sources of community support and incorporate them into the care network for the patients.

After the consultation, it is important to undergo debriefing. Employee assistance practitioners are available in many health facilities across the country for this service. However, debriefing can be carried out by a fellow colleague. Clinicians must assess their own feelings of the experience with the patients with COVID-19. This will assist clinicians to reset their emotional state to ‘neutral’ in order to prepare for the next patient sensitively, without carrying over the negative encounter in one consultation to another.
